# Antimicrobial stewardship interventions currently implemented at primary healthcare settings across low- and lower-middle-income countries (LLMICS)

**DOI:** 10.1093/inthealth/ihaf136

**Published:** 2026-01-22

**Authors:** Abdulhammed O Babatunde, Abdulmumin Damilola Ibrahim, Oluwaseyi M Egbewande, Wuraola Olabode, Nabeelah Aroyehun, Warittha Tieosapjaroen, Yusuf Babatunde, Eneyi Kpokiri

**Affiliations:** Faculty of Clinical Sciences, College of Medicine, University of Ibadan, 200005 Ibadan, Nigeria; Faculty of Pharmaceutical Sciences, University of Ilorin, Ilorin, Nigeria; Faculty of Pharmaceutical Sciences, University of Ilorin, Ilorin, Nigeria; Faculty of Pharmaceutical Science, University of Lagos, Lagos, Nigeria; Medicine and Surgery, University of Ilorin, 240003 Ilorin, Nigeria; School of Translational Medicine, Faculty of Medicine Nursing and Health Sciences, Monash University, 3800 Melbourne, VIC, Australia; Faculty of Pharmaceutical Sciences, University of Ilorin, Ilorin, Nigeria; Clinical Research Department, Faculty of Infectious and Tropical Diseases, London School of Hygiene and Tropical Medicine, WC1E 7HT London, UK

**Keywords:** antimicrobial stewardship, AMR, primary healthcare, ASP, LLMICs

## Abstract

**Background:**

Antimicrobial resistance (AMR) is a top global public health and development threat. Antimicrobial stewardship programs (AMSPs) are one of the most cost-effective interventions to optimize the use of antimicrobials. This study reviews AMSPs that have been implemented in low- and lower middle-income countries.

**Methods:**

A systematic search was conducted on electronic databases including MEDLINE, PubMed, Embase, OVID, Web of Science and Cochrane Library on 18 July 2024 for published papers from 2014 to 2024 following the Preferred Reporting Items for Systematic Reviews and Meta-Analyses guideline. Relevant published literature was then selected based on the established inclusion/exclusion criteria. Each article was screened by two independent reviewers. Data were extracted and synthesized in the review.

**Results:**

Of the 425 articles screened, only 13 were eligible for review and included in this study. Two studies were multinationals. Five studies were randomized controlled trials. Among the three key focuses of AMSPs, most of the interventions focused on optimizing antibiotic use (n=8), followed by improving diagnostics and monitoring (n=3) and education and training (n=2). The most commonly reported barriers to implementing AMSPs was a lack of resources (n=9). Facilitators reported included knowledge of AMS (n=8), availability of educational and training resources (n=8), adequate funding (n=6), accountable and transparent procedures (n=5) and positive communication within healthcare facilities (n=4).

**Conclusions:**

All included studies show improvement in AMS through innovative programs. However, only a few have been adopted nationwide and influence policy formulation in the country. We recommend adoption of effective AMSPs into the national strategic planning and implementation across primary health settings.

## Introduction

Antimicrobial resistance (AMR) is a top global public health and development threat. The first comprehensive analysis of the global impact of antimicrobial resistance (AMR) estimated that resistance caused approximately 1.27 million deaths in 2019 and that antimicrobial-resistant infections played a role in about 4.95 million deaths. Estimates for 204 countries and territories confirm AMR as a global health threat, with the worst impacts in low- and middle-income countries (LMICs), although higher-income countries also face alarmingly high levels of AMR.^1^

Antimicrobial stewardship programs (AMSPs) are one of the most cost-effective interventions to optimize the use of antimicrobial medicines, improve patient outcomes and reduce AMR and healthcare-associated infections (HAIs). Good antimicrobial stewardship ensures the optimal selection, dose and duration of an antimicrobial therapy that leads to the best clinical outcome for the treatment or prevention of infection while producing the fewest toxic effects and the lowest risk for subsequent resistance.^2^ The purpose of the AMSP is to ensure the proper use of antimicrobials within the healthcare system through the development of a formal, interdisciplinary team. The primary goal of the AMSP is to optimize clinical outcomes while minimizing unintended consequences related to antimicrobial usage, such as toxicities or the emergence of resistance.^3^

In LMICs, the determinants of antibiotic prescribing practices are wide-ranging.^4^ Studies with health professionals have identified a poor appreciation of core principles, knowledge of antibiotic prescribing and problems of resistance, limited continuing medical education, a lack of updated policies and treatment guidelines, the quality of antimicrobial medicines and selective pressures from pharmaceutical companies. There is commonly limited availability of expertise and diagnostic facilities to guide the choice of antibiotics.^5^

Primary care is critical to the overall functioning of healthcare systems globally, especially in low- and lower-middle-income countries (LLMICs). In these regions, primary care serves as the first point of contact for most patients, playing a crucial role in preventing, diagnosing and treating a wide range of health conditions. It also ensures equitable access to essential health services, particularly for underserved and rural populations. This pivotal role has been emphasized in various studies and reports, highlighting its importance in addressing the health needs of populations in LMICs. A study by Kruk et al.^6^ emphasizes that well-functioning primary care systems improve access to essential health services and reduce the burden on secondary and tertiary care facilities, which are often less accessible in rural or underserved areas. This is especially important in LLMICs, where distance and financial constraints can prevent individuals from accessing higher levels of care.^6^

Primary care providers in LLMICs face a wide range of systemic challenges, including limited resources, inadequate training, weak regulatory frameworks and the unregulated sale of antibiotics. These challenges hinder the ability of primary care systems to deliver high-quality, equitable healthcare and contribute to wider public health problems, such as the rise of antimicrobial resistance. Addressing these issues requires substantial investment in infrastructure, workforce development, regulatory reforms and public education to improve healthcare outcomes in LLMICs.^7^

AMSPs have been highly successful in high-income countries (HICs) in improving antibiotic use and combating AMR. These programs focus on optimizing antibiotic prescribing practices to enhance patient outcomes, reduce inappropriate use and minimize the emergence of resistant bacteria. A systematic review by Baur et al.^8^ concluded that AMSPs effectively improve compliance with recommended antibiotic use in hospitals, reducing unnecessary broad-spectrum antibiotic prescriptions.

A scoping review by Charani et al.^9^ highlighted the diversity of implementation strategies for AMSPs in different settings, yet few studies have specifically focused on their applicability in primary care settings within LLMICs. Harun et al.^10^ examined the current state of hospital-based AMSPs in LMICs, shedding light on barriers, facilitators, prescribers’ perceptions and practices and the impact of ASP interventions.

Moreover, while reviews such as those by Baur et al.^8^ and Ababneh et al.^11^ have established a solid foundation regarding AMSPs in hospitals of HICs and the Middle East, respectively, there remains a notable absence of systematic assessments of AMSPs in primary healthcare (PHC) settings of LLMICs. This gap is critical to address, as primary care is often the first point of contact for patients and plays a vital role in antibiotic prescribing practices. The five essential public health functions in PHC include health protection, health promotion, disease prevention (service delivery), surveillance and preparedness (intelligence). These functions are key to effective prevention and management of infection as well as more appropriate use of antibiotics, which underpins AMR control.

This study reviews AMSPs that have been implemented in LLMICs. It is essential to map the current evidence base, identify gaps in knowledge and explore contextual challenges unique to LLMICs.

## Methods

### Scoping review framework

We organized a scoping review of the literature, drawing on the framework of Arksey and O’Malley^12^ with some modifications from Levac et al.^13^ and the Preferred Reporting Items for Systematic Reviews and Meta-Analyses (PRISMA) extension for scoping reviews. The scoping review consisted of the following five stages: identification of a research question, identification of relevant articles, selection of articles, data charting and collating, summarising and reporting the results.

### Search strategy

A systematic search was conducted on electronic databases including MEDLINE, PubMed, Embase, OVID, Web of Science and Cochrane Library on 18 July 2024. An extensive search was also performed using the reference lists of relevant articles and national reports on AMR. We also conducted a secondary search on 3 August 2024 to capture any new articles published during the year after our initial search. The search algorithm included variations of the following terms: antimicrobial, stewardship, programmes, primary care setting and LLMICs.^14^

### Inclusion and exclusion criteria

Relevant published literature was then selected based on the established inclusion and exclusion criteria. We included publications reporting primary data on AMS interventions implemented in PHC settings across LLMICs. The search was restricted to interventions in humans and studies published in the English language between January 2014 and June 2024. Only full manuscripts were included. Conference abstracts were excluded because of inadequate details on the AMSPs implemented. Reviews, commentaries/editorials, opinion pieces and primary study protocols that described potential programs that have not been implemented were excluded.

### Study selection

Four reviewers (IA, OE, WO and NA) independently screened titles and abstracts. Any discrepancies were resolved by two reviewers (AOB and WT). Five independent reviewers (IA, OE, WO, NA and AOB) screened full-text manuscripts. Data were extracted to provide relevant answers to the research questions: ‘What are the AMS interventions currently implemented at the PHC settings across LLMICs?, ‘What are the barriers and facilitators to implementing antimicrobial stewardship programs in LLMICs?’, and ‘What are the current gaps and areas of improvement of these AMS interventions?’. Subsequently, a narrative synthesis of the extracted data was performed. The stages of our narrative synthesis included descriptive statistics to summarize the extent and nature of included studies and thematic categorization, which involved identifying common training areas and grouping studies based on those training categories.

### Data charting process

**Figure 1.**
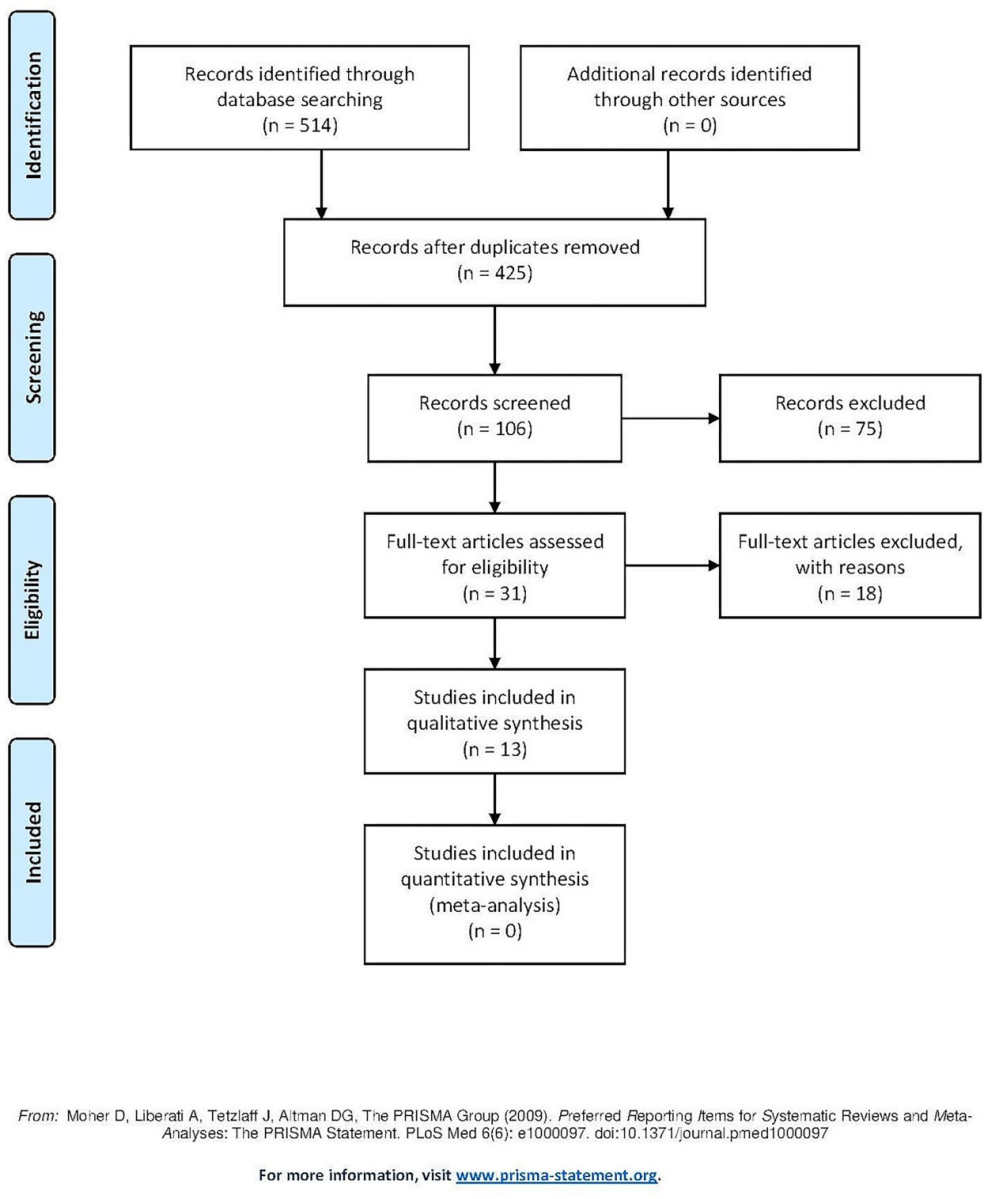
Adapted PRISMA flowchart summarizing the search and selection of studies.

The 2009 PRISMA tool adapted from Moher et al.^15^ was used to map the relevant articles selected (Figure 1). Based on an adapted PICOS framework (population, intervention, comparison, outcomes and study setting), a standardized data extraction sheet was used to guide the eligibility of studies selected, containing the following fields: author, year, study design, study location, study population, aims of the study, intervention and summary of results. The Mixed Method Appraisal Tool version 2018^16^ was used to assess the quality of the included studies.

## Results

Of the 425 articles screened, only 13 were eligible for review and included in this study (Table 1). Two studies were multinational while others were from Ghana (n=2), Kenya (n=2), Tanzania (n=2), Vietnam (n=2), Uganda (n=1), Burkina Faso (n=1) and India (n=1). Most of the studies were published from 2020 to 2024 (n=8). Five studies were randomized controlled trials (RCTs). Other study designs were qualitative (n=3), cross-sectional (n=2), open call/case series (n=1), pre and post cross-sectional (n=1) and observational (n=1). Among the three key focuses of AMSPs, most of the interventions focused on optimizing antibiotic use (n=8), followed by improving diagnostics and monitoring (n=3) and education and training (n=2). A total of 10 studies had a score of 4 or 5 from the quality assessment.

**Table 1.** Summary of all included studies and key findings.

Author	Publication year	Country of study	PHC setting	Aim of study	Study design	Study population	Intervention	Participants/sample size, n	Key findings
Cundill et al.^25^	2015	Tanzania	Primary health centre	To improve adherence to national and WHO malaria diagnosis and treatment guidelines	RCT	Patients	Prescribers from facilities in the HW and HWP had small interactive peer-group training sessions, with the HWP additionally receiving clinic posters and patient leaflets. Performance feedback and motivational mobile phone text messaging (SMS) were added to the HW and HWP arms in later phases	44 121 eligible patients	Standard RDT training reduced pretrial levels of antimalarial prescribing, which was sustained throughout the trial. Both interventions significantly lowered incorrect prescribing of recommended antimalarials from 8% in the standard training arm to 2% in the HW arm (p=0.008) and 2% in the HWP arm (p=0.005)
Do et al.^24^	2016	Vietnam	Primary health centre	To evaluate point-of-care CRP testing to reduce inappropriate use of antibiotics for non-severe acute respiratory infections in Vietnamese PHC	RCT	Patients	Implemented CRP POCT before prescribing antibiotics for patients with non-severe acute respiratory tract infections in Vietnam	2037 patients	This study shows that access to CRP POCT reduces unnecessary antibiotic use for non-severe acute respiratory infections in adults and children in PHC in Vietnam without compromising clinical recovery or serious adverse events. 64% of patients used antibiotics within 14 d in the CRP group versus 78% in the control group (OR 0.49 [95% CI 0.40 to 0.61], p<0.0001) Highly significant differences were seen in both children and adults, with substantial heterogeneity of the intervention effect across the 10 sites (I^2^=84% [95% CI 66 to 96])
Kleczka et al.^26^	2019	Kenya	Primary health centre	To demonstrate improved documentation of care for common infectious conditions	Qualitative study	10 facilities	The intervention included four elements: rubber stamp templates for documenting management of selected conditions, compilations of the relevant clinical practice guidelines, one low-budget (≈$70) Android smartphone to each facility and one continuing medical education session at each facility every month	10 facilities	Completeness of chart documentation improved slightly but significantly from 33.0±1.0% pre-intervention to 38.3±1.4% post-intervention (t=−3.09, p<0.01). The greatest improvements in documentation scores were seen when templates were used. Overall template documentation scores (69.5±1.7%) were significantly higher than both pre-intervention charts (t=−19.61, p<0.00001) and post-intervention charts (t=−14.28, p<0.00001). Template scores were significantly higher than post-intervention charts across all dimensions of documentation—general patient information
Kandeel et al.^28^	2019	Egypt, United Arab Republic	Primary health centre	To describe antibiotic prescribing practices for acute respiratory infections and knowledge and attitudes of physicians, pharmacists, and patients before and after a multidimensional behaviour change strategy	RCT	Healthcare workers, patients	An intensive campaign to promote appropriate antibiotic prescription was launched. Multiple focus group discussions were conducted with physicians, pharmacists, non-governmental organizations and the general public in order to define the key messages and potential communication channels. A 5-d training course by an international infectious disease consultant was held to educate participants on appropriate management of acute respiratory infections	41 PHC units	There was a 25% decrease overall in antibiotic prescribing post-intervention for children from 82.1% to 61.5%; the largest improvements in prescribing in these patients were for ear infection and the common cold. There was a 22% decrease overall in antibiotic prescribing for adults from 86.7% to 67.9%, which was driven by large changes in prescribing for patients with ear infections and bronchitis
Hadley and Beard^21^	2019	Tanzania	Primary health centre	To determine the impact of PBF with direct quality indicators on reducing overprescription of antibiotics in primary care settings	Observational	Health workers, patients	The PBF model involved making payments to health facilities and their staff for specific services, with additional ‘direct quality indicators’ attached to some of these services. It included payments for 18 pre-agreed services, 9 of which had additional direct quality indicators attached. The study evaluates the impact of this intervention on antibiotic use and adherence to treatment guidelines in primary care settings	56 health facilities	The introduction of PBF with ‘direct quality indicators’ for adherence to treatment guidelines significantly improved the proportion of patients treated according to guidelines, increasing from 25.5% to 85.7% over the pilot period. PBF also led to a substantial reduction in the proportion of patients prescribed antibiotics not in accordance with treatment guidelines, from 39% down to 2–6% in the PBF districts. The improvements in antibiotic prescribing were sustained throughout the 3-y pilot period in the PBF districts
Balakrishnan et al.^44^	2020	India	Primary health centre	To assess the impact of the lung health care project on the consumption of CRD drugs and prescription practices	Qualitative study, mixed methods	Healthcare workers (doctors), patients	Standard steps of pilot implementation were followed from estimating the burden of CRDs, assessing the capabilities of the health infrastructure, developing and testing technical and operational guidelines, designing communication messages, formulating an information system, developing training materials and training of staff	196 CRD patients	There was reduction in the use of oral antibiotics and injectable bronchodilators and steroids in the LHCP and control institutions. The reduction in antibiotic prescription in control institutions during the concurrent period was 13.4%. Injections (a bronchodilator and/or steroids) were prescribed for 37 (39.4%) CRD patients at baseline in the LHCP institutions, while 20 (20%) patients had the same after 6 months (p=0.002) showing a reduction of 49.24%. The control institutions showed a reduction of 13.4% (p=0.269)
Peiffer-Smadja et al. ^22^	2020	Burkina Faso	Primary health centre	To examine the requirements for a CDSS adapted to the context of primary care in West Africa and to analyse the barriers and facilitators of its implementation and adaptation by prescribers using qualitative methods during a pre-implementation workshop	Qualitative study	Healthcare professionals	The intervention was a 3-h workshop that presented the antibiotics CDSS for antibiotic prescribing in primary care to 47 healthcare professionals from nine West African countries, followed by a roundtable discussion and completion of a questionnaire to gather feedback on the potential implementation and adaptation of the CDSS in the West African context	47 participants	The use of a CDSS for antibiotic prescribing (and other clinical decisions, in fact) is currently missing, but the importance and need for such tools to support antibiotic prescribing in primary care were stressed by all participants. Regarding the implementation of a CDSS in primary care, the participants encouraged a procedure of co-designing the tool with health professionals and stakeholders involved in antibiotic prescribing and initiation via a primary care pilot site, which is linked to an academic hospital
Basu et al.^23^	2021	Bolivia, Ethiopia, India, Kenya, Malawi, Mauritania, Nigeria, Philippines, Tanzania	PHC, tertiary care etc	To identify real-world solutions to problems and to determine whether there were broader lessons from such innovations	Case series (open call)	Clinicians	A broader community intervention with educational talks to mothers, focused on the long-term health risks of antibiotic overuse. The mothers were supported to adhere to medical advice through laboratory confirmation of infection where possible	NA	The infant was effectively treated with 5% permethrin cream and cessation of antibiotics. His mother was counselled on the adverse effects of antibiotics
Nyamu et al.^27^	2021	Kenya	Community health centre	To address the main health needs of the community and to build the capacity of healthcare workers in the primary care clinics in that area	Cross-sectional	Family medicine residents, patients	An interactive educational intervention with the health facility staff and CHVs was done to highlight the burden of URTIs as the most common presenting ailment in the health facilities. The educational sessions were fully interactive, exploring understanding of the staff on AMR, factors influencing the high number of clinical visits for URTIs and the reasons for the high antimicrobial usage. A presentation on the management of URTIs, based on the Kenyan guidelines, was made with a discussion on evidence-based management	3014 patients	Antimicrobial prescription in the <5-y age group was reduced by 44% in the 2 weeks following the intervention and by 18% at weeks 8–9. In the >5-y age group, prescribing was reduced by 18% and 8%, respectively
Do et al.^17^	2023	Vietnam	Primary health centre	To assess whether introduction of pointof-care CRP tests in routine PHC could safely reduce prescription of antibiotics for patients with acute respiratory infections	RCT	Patients	A cluster-randomised trial of POCT of CRP in patients with acute respiratory infections in PHC in Viet Nam	39 856 point-of-care test	It was shown that use of point-of-care testing for CRP concentrations in people with suspected acute respiratory infections reduced initial antibiotic prescribing in PHC centres in Viet Nam without compromising clinical recovery. There was substantial heterogeneity in prescription reductions before and during intervention between the CHCs (I^2^=98.7% [95% CI 97.5 to 99.1], p<0.0001)
Kiggundu et al.^19^	2023	Uganda	Primary health centre	To describe the implementation and evaluation of CQI approaches for AMS in six hospitals in Uganda	Pre and post cross-sectional	Hospital AMS teams	Using the WHO AMS toolkit to set up hospital AMSPs and implemented interventions using CQI techniques and targeting conditions commonly associated with antibiotic misuse. The interventions included training, mentorship and provision of clinical guidelines to support clinical decision-making	NA	There was an overall reduction in antibiotic use after the interventions for both UTIs and URTIs. However, this reduction was observed to be greater for UTIs. There was a 20.7% reduction in the mean number of antimicrobials per patient for URTIs from the pre-intervention to the intervention phase, from 0.8 to 0.6, respectively (p<0.001), and a reduction in the number of treatment days (p=0.0163). There was a 19.2% overall reduction in the mean number of antimicrobials per patient for UTI from the pre-intervention to the intervention phase (p<0.01)

HW: health worker; HWP: health worker-patient; OR: odds ratio; CI: confidence interval; CRD: chronic respiratory disease; LHCP: lung health care project; CDSS: clinical decision support system; CHV: community health volunteers; URTI: upper respiratory infection; CHC: commune health centres; UTI: urinary tract infection.

### Quality assessment

In the scoping review, 13 studies were examined to identify AMSP strategies implemented in PHC settings across LLMICs. Each study was assessed using a standardized quality assessment tool with scores ranging from 1 to 5, where higher scores indicate better methodological quality. The quality assessment revealed a generally high quality of study design and methodology, with 9 of the 13 studies scoring 4 or 5. This is an indication that most of the studies reviewed are of strong to moderate strong quality, providing reliable evidence for the AMS interventions.

### Interventions to optimize antibiotics use

Eight studies in India, Burkina Faso, Tanzania, Uganda and Vietnam reported AMSPs that focused on optimizing antibiotic use, such as improving prescribing practices, reducing unnecessary antibiotic use and developing and implementing guidelines. Two studies^17,18^ reported the positive impact of point-of-care testing (POCT) in PHC centres for common diseases such as respiratory tract infections in children. POCT for C-reactive protein (CRP) significantly reduced antibiotic prescriptions in Vietnam primary care centres (p<0.0001).^17^ POCT packages were used in Ghana district hospitals to distinguish pathogenic from non-pathogenic causes of febrile illness among community members.^18^ Two studies^19,20^ implemented the standardized World Health Organization (WHO) tool kit for AMS in a primary care setting to optimise antibiotics usage.^19^ Implemented interventions using continuous quality improvement (CQI) techniques significantly reduced antibiotics usage in Uganda (p<0.001) and targeted conditions commonly associated with antibiotic misuse.^19^ Implementation of performance-based financing (PBF) with direct quality indicators among health workers in Tanzania improved adherence to standard guidelines for AMS from 25.5% to 85.7%.^21^ Co-creation of AMSPs was described by two studies.^22,23^ However, they have not been evaluated for effectiveness.

### Interventions to improve diagnostics and monitoring

Only three studies focused primarily on improving diagnostics and monitoring, such as enhanced laboratory capabilities, promoting accurate diagnosis and monitoring and reporting antibiotic use and resistance. Studies by Do et al.^24^ and Cundill et al.^25^ showed that implementing POCT in PHC centres significantly reduced unnecessary use of antimicrobials among 39 856 and 44 121 participants in Vietnam and Tanzania, respectively. Intervention to improve monitoring of antibiotics use in treating common infections in primary care settings was implemented in Kenya.^26^ The study AMSP used rubber stamps and a data collection template as a low-cost strategy to improve documentation of antibiotics used in managing infections in the PHC centres.^26^

### Education and training

Only two studies focused primarily on education and capacity-building interventions of PHC professionals and the community to improve AMS. The provision of education on the causes and burden of AMR and the need to control antimicrobial usage through interactive education workshops for community health workers showed a reduction in the rate of antibiotic prescriptions in children <5 y of age presenting at community health centres.^27^ Kandeel et al.^28^ conducted RCTs to explore the effect of a community campaign and education involving health professionals, community members and other stakeholders. This resulted in a 25% and 22% decrease in antibiotics prescriptions for children and adults, respectively, at 41 PHC centres.

### Barriers and facilitators to implementing AMSPs

**Figure 2.**
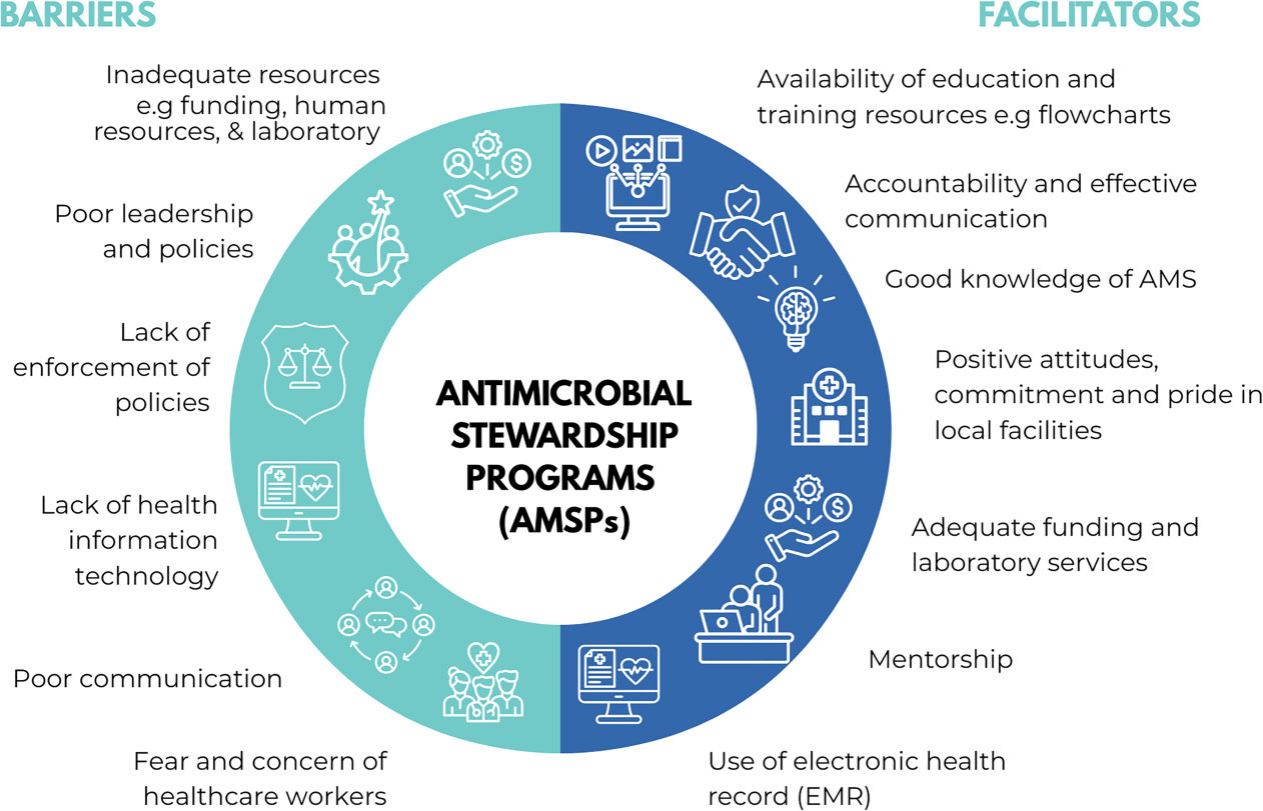
Barriers and facilitators of AMSPs.

The most commonly reported barriers to implementing AMSPs were a lack of resources (n=9), a lack of adequate knowledge, education and training of AMS (n=5), inadequate laboratory services (n=4) and a lack of enforcement of policies (n=4). Facilitators reported included knowledge of AMS (n=8), availability of educational and training resources (n=8), adequate funding (n=6), accountable and transparent procedures (n=5) and positive communication within healthcare facilities (n=4) (Figure 2).

### Meta-analysis of RCTs

Six RCTs were included in the meta-analysis, with a mean effect size of −1.3082 (standard error 0.2982), p-value <0.001, heterogeneity 96.57% and τ^2^ 0.4486 (Table 2).

**Table 2.** Meta-analysis of RCTs included in the review.

	Treated			Control			
Study	Events	Non-events	Total n	Events	Non-events	Total n	OR
Cundill et al.^25^	7	1600	1607	106	1191	1297	0.0492
Adjei et al.^18^	294	466	760	323	422	745	0.8243
Do et al.^17^	17 345	1276	18 621	20 860	375	21 235	0.2444
Balakrishnan et al. 2020^44^	21	18	39	36	2	38	0.0648
Kandeel et al.^28^	391	216	607	277	54	331	0.3529
Do et al.^24^	581	321	902	738	209	947	0.5126
**Random effects statistics summary**							
**Mean effect size**	−1.3082	**Variance**	0.0890	**Standard error**	0.2982		
95% confidence interval	−1.8927	−0.7236					
**Z**	−4.3862	**p-value**	<0.001				
**Heterogeneity I^2^**	96.57%	(97.85%, 94.51%)		**τ** ^2^	0.44860		

## Discussion

This scoping review examined the available literature on AMSPs for primary care settings in LLMICs. Most of these studies were published between 2020 and 2024, reflecting the growing attention to AMS in LMICs in recent years. This study design, while broad, is an indication of the challenges of conducting rigorous trial studies in LLMICs due to limited resources. Hence this often leads to reliance on observational and cross-sectional data.

The majority of the studies focused on optimizing antibiotics use, which is appropriate and critical, seeing the high rates of antibiotics misuse in LLMICs which can be attributed to factors like limited access to diagnostic tools, poor prescribing practices and a lack of robust policies.^29^ The remaining studies demonstrated a limited focus on improving diagnostics and education, although these areas are equally vital to sustainable AMS efforts.^30,31^

The most commonly reported barrier was a lack of resources, with nine studies emphasizing it. Other barriers reported include a lack of adequate knowledge, education and training, inadequate laboratory services and a lack of policy enforcement. These findings align with an earlier study where substandard laboratory facilities, inadequate human resources and a lack of training were documented as impediments to AMSPs, among others.^10,32^ The scoping review also reported a number of facilitators to AMSP implementation in LLMICs, including knowledge of AMS, adequate funding, accountable and transparent procedures and positive communication within healthcare facilities. These findings are in line with the recommendations of the WHO on improving the knowledge, education and training of health professionals on appropriate antibiotics use on the road to implementing AMSPs.^31^

### AMS interventions in PHC settings across LLMICs

AMS interventions in LLMICs often focused primarily on optimizing antibiotic use, with fewer addressing diagnostics, monitoring and education. However, this is in contrast with high-income countries where AMS interventions focus more on advanced diagnostics and monitoring systems due to better resource availability.^33^

### Interventions to optimizing antibiotic use

The basic focus of antimicrobial interventions in LLMICs is on optimizing antibiotic use and reducing misuse, which is an essential step in curbing inappropriate prescribing practices. Eight studies reported interventions targeting antibiotic prescribing behaviours, reducing unneeded antibiotic use and developing and/or enforcing clinical guidelines. This is in line with the findings of Tinker et al.^34^ in their study on interventions to optimize antimicrobial stewardship. A number of strategies have been implemented in PHC settings across LLMICs, including India, Burkina Faso, Tanzania, Uganda and Vietnam. Two of the studies^17,18^ showed the positive impact of POCT for CRP in PHC centres in Vietnam (p<0.0001) and Ghana, significantly reducing antibiotic prescriptions for respiratory infections in children and adults. This is an indication of the importance of POCT in settings where diagnostic uncertainty often leads to overprescription or inappropriate prescription of antibiotics. This is further supported by the findings of Llor et al.^35^ in their study in Europe that reported CRP POCT has proven to be effective in safely reducing the use of antibiotics and is considered to be the best available option to combat AMR in primary care.

Similarly, the WHO toolkit for AMS was adopted and implemented in Uganda and Ghana, where its use has led to remarkable reductions in antibiotic use.^17,18^ This can be seen in the study by Kiggundu et al.^19^ in Uganda, where they implemented interventions using CQI techniques that significantly reduced antibiotic use (p<0.001). In another study by Hadley and Beard^21^ in Tanzania, PBF was linked to AMS adherence, thereby increasing healthcare worker’s compliance with AMS guidelines from 25.5% to 85.7%. This shows how structured AMS interventions, if and when they are properly implemented, can be effective in optimizing antibiotic use in LLMICs. Despite the fact that these interventions have shown success, evidence suggests that scale-up remains challenging due to infrastructural constraints. Although two of the studies^22,23^ reported co-creation of AMSPs, they have not been evaluated for effectiveness. However, in another study conducted in sub-Saharan Africa using a co-creation approach, it revealed that co-created AMS tools are necessary for managing antimicrobial use across healthcare settings and increasing local AMS ownership and commitment.^36^

### Interventions to improve diagnostics and monitoring

Only three studies focused primarily on interventions to improve diagnostics and monitoring.^24–26^ Advanced laboratory capabilities and accurate diagnosis are essential to reducing unnecessary antibiotic use, as can be seen in the study by Do et al.,^24^ where access to CRP POCT in Vietnam was able to reduce antibiotic use in PHC centres. Llor et al.^35^ further confirm the significance of advanced diagnostic capacities in reducing antibiotic misuse through CRP POCT. Cundill et al.^25^ reported a similar impact in Tanzania, which further reinforced the importance of access to diagnostics in low-resource settings. Several global studies have also recognized diagnostics as having a broad impact on multiple areas of AMSPs and in reducing AMR.^37–39^ In addressing monitoring of antibiotics used in treating common infections in primary care settings, an innovative low-cost strategy was introduced in Kenya using rubber stamps and a data collection template to improve the documentation of antibiotic use in PHC centres, thereby providing a simple solution to challenges posed by poor documentation.^26^ However, the limited and underrepresented diagnostics-focused interventions is indicative of a gap in this area and this could be attributed to the limited availability of diagnostic tools and laboratory infrastructure in many LLMICs. Improving diagnostics and monitoring is critical for improving treatment accuracy and reducing antibiotic misuse, specifically in settings where broad-spectrum antibiotics are used regularly due to uncertainty.

### Interventions aimed at education and training

Of the 13 studies, only 2 focused on education and training for health workers, patients and the community to improve AMS. Education is key in improving AMS, as increasing awareness of antibiotic resistance among healthcare workers and the community is critical for fostering better antibiotic use practices. Nyamu et al.^27^ found that interactive education workshops for community health workers could reduce the rate of antibiotics prescriptions in children <5 y of age. Similarly, Kandeel et al.^28^ noted a respective 25% and 22% decrease in antibiotics prescriptions for children and adults at PHC centres after consecutive community campaigns and training involving the health professionals and community members. However, the review revealed that there is limited emphasis on education and training interventions in LLMICs. This is a critical gap, as inadequate knowledge and awareness are frequently reported as barriers to effective AMS implementation.^32^ These gaps could be addressed through public education and training programs, which will consequently bolster AMS efforts in these settings.

### Do policies/guidelines support AMS interventions in LLMICs

The scoping review showed that some policies and guidelines were put in place in certain locations, such as the use of WHO toolkits in Uganda and Tanzania. However, policy support for AMS interventions across LLMICs remains inconsistent. Despite this, the reviewed studies showed that integration of policies into primary care can significantly improve antibiotic use and reduce misuse. This aligns with the findings of Mpundu,^40^ which emphasized the development of national action plans on AMR to support AMS interventions. The WHO toolkit provides guidance on strategies for the development of AMSPs in LLMICs.^41^ Likewise, institutional policies are one of the AMS strategies employed to improve antibiotics use in LLMICs, as seen in the study by Cox et al.^42^ However, the lack of strong policy frameworks and political will in many LLMICs has often resulted in uneven implementation of these interventions. Additionally, the lack of national AMS policies and a weak regulatory framework tend to create significant challenges for the sustainability of AMS interventions, as observed by Sefah et al.^20^ It is established that policies and guidelines can and have supported AMS interventions when present. As such, strengthening this alongside consistent application of guidelines across LMICs will be essential in building more resilient AMS systems.

### What are the barriers and facilitators to implementing AMSPs in LMICs?

Several factors have been reported as barriers to the implementation of AMSPs in LMICs. The most commonly reported barriers were a lack of resources, a lack of adequate knowledge, education and training of AMS, inadequate laboratory services and a lack of enforcement of policies. These findings have also been reported among similar studies conducted in high-resource settings. For instance, a study conducted at Ministry of Health hospitals in Saudi Arabia reported by Alghamdi et al.^43^ revealed poor communication, a lack of recruitment/a shortage of ASP team members, a lack of education and training and a lack of health information technology as major barriers to the implementation of AMSPs. This suggests that the barriers to AMS are not limited to financial restrictions or economic settings. Therefore, a holistic approach is required to effectively overcome the barriers to AMS. This approach must also centre on fostering behavioural change among healthcare professionals, patients and communities.

The facilitators of AMSPs, such as knowledge of AMS, availability of educational and training resources, adequate funding, accountable and transparent procedures and positive communication within healthcare facilities, must also be considered and prioritised in the process of improving the execution of AMSPs.

The meta-analysis showed a high mean effect size (−1.3082) and p<0.001, indicating the effectiveness of the AMS interventions in reducing antibiotics prescriptions and use in PHC facilities. However, the high heterogeneity suggests wide variations in the RCTs included in the meta-analysis.

### What are the current gaps and areas of improvement of these AMS interventions?

The AMS interventions did not describe the cost analysis of the programs or interventions to determine the cost-effectiveness and sustainability of interventions. Also, there is no framework or policy for AMS interventions specific to primary care settings. The interventions mostly focus on hospital settings. However, there is a need for more community-based interventions to control self-medication and other mechanisms of antimicrobial abuse, such as among livestock farmers. There is a need for behavioural-based interventions and sustained community awareness programs. Also, interventions that show significant improvement in AMS have not been adopted by the government or agencies focusing on AMR to support scalability and increase impact. There is a need for more interventions that support diagnosis and monitoring in LLMICs. More funding to strengthen AMS in LLMICs should focus on equitable access to POCTs and the establishment of data collection and management systems on AMR. Also, there are few publications describing AMS interventions in LLMICs, which shows the need for more innovative interventions in other LLMICs, especially in countries with the highest burden of AMR.

### Limitations

This study has some limitations. First, the search term and inclusion criteria only included studies published in the English language. Hence we might have excluded some AMSPs published in other languages. Also, we excluded grey literature, conference papers and other papers without a full manuscript available explaining the AMSP implemented. Lastly, we only reported human studies, thereby excluding animal-based studies, which also contribute significantly to AMR in primary care settings. The generalizability of this review may be limited given the scope, but there are important lessons to be gleaned from the varied interventions in diverse LLMICs included in the review.

## Conclusions

This scoping review summarises the current state of AMSPs in PHC settings across LLMICs. All included studies show improvement in AMS through innovative programs. However, only a few have been adopted nationwide and influence policy formulation in the country. The findings demonstrate a significant focus on interventions to optimize antibiotic use, while diagnostics, monitoring and educational efforts remain underrepresented. Barriers and facilitators of successful implementation have also been highlighted. We recommend adoption of effective AMSPs into the national strategic planning and implementation across PHC settings.

## Data Availability

There are no new data associated with this article.
